# Diagnostic Utility of PRAME Expression in Melanocytic Lesions: Cut-Off Threshold Analysis

**DOI:** 10.3390/diagnostics15202595

**Published:** 2025-10-15

**Authors:** Beste Noyan Mod, Cem Leblebici

**Affiliations:** 1 Department of Pathology, Basaksehir Cam and Sakura City Hospital, University of Health Sciences, 34480 Istanbul, Turkey; 2 Department of Pathology, Istanbul Training and Research Hospital, University of Health Sciences, 34098 Istanbul, Turkey; cleblebici@gmail.com

**Keywords:** PRAME, melanoma, immunohistochemistry, melanocytic lesions

## Abstract

**Background/Objectives**: PRAME (Preferentially Expressed Antigen in Melanoma) is a promising immunohistochemical marker for distinguishing melanoma from benign melanocytic lesions, though optimal thresholds remain uncertain. This study evaluated PRAME expression in melanocytic lesions and compared diagnostic accuracy using two thresholds. **Methods**: We retrospectively assessed PRAME expression in 145 melanocytic lesions diagnosed between 2016 and 2021 at Istanbul Training and Research Hospital: 52 melanomas, 27 dysplastic nevi, 23 Spitz nevi, 15 compound nevi, 23 blue nevi, and 5 congenital nevi. Immunohistochemical staining (PRAME EP461, Cell Marque) was scored semi-quantitatively based on nuclear positivity: 0 (negative), 1 (1–24%), 2 (25–49%), 3 (50–74%), and 4 (≥75%). Diagnostic accuracy was evaluated at 50% and 75% thresholds. **Results**: PRAME expression at both thresholds was significantly higher in melanomas than nevi (*p* < 0.05). Sensitivity and specificity were 92.3% and 96.8% at 50%, and 82.7% and 98.9% at 75%. Lowering the threshold to 50% improved sensitivity with minimal specificity loss, particularly differentiating melanoma from dysplastic, compound, and blue nevi. Occasional positivity was observed in Spitz and dysplastic nevi; one melanoma was PRAME-negative. **Conclusions**: PRAME is an effective marker for melanoma diagnosis. A 50% threshold optimizes sensitivity while preserving specificity; however, histopathological evaluation remains the gold standard, and PRAME should be used only as an adjunct to avoid potential overdiagnosis, particularly in borderline lesions.

## 1. Introduction

Melanomas are malignant tumors derived from melanocytes and represent one of the most aggressive forms of skin cancer. Although they account for less than 5% of cutaneous malignancies, they are responsible for the majority of skin cancer-related deaths worldwide [1,2,3]. Global incidence has been steadily rising, particularly in fair-skinned populations, with approximately 160,000 new cases and 48,000 deaths reported annually [4]. Despite recent advances in targeted therapies and immunotherapy that have improved survival rates [5], early and accurate diagnosis remains critical for patient management.

Histopathological examination continues to be the gold standard for melanoma diagnosis [6]. However, in morphologically ambiguous lesions with overlapping benign and malignant features, diagnostic uncertainty still persists [7]. Immunohistochemical markers such as S-100, HMB-45, Melan-A, Ki-67, and p16 are widely used to support diagnosis, but none provide absolute specificity or sensitivity [8,9]. Given these limitations, novel biomarkers with higher diagnostic accuracy are of particular interest, among which PRAME has emerged as a promising candidate.

First described by Ikeda et al. in 1997, PRAME is classified as a cancer/testis antigen (CTA) encoding the human leukocyte antigen (HLA)-A24 antigen [10]. The PRAME gene is located on the antisense strand of chromosome 22 (22q11.22), spans approximately 12 kilobases, and contains leucine-rich repeat regions [11]. PRAME can be detected in various malignancies such as melanoma, acute leukemia, and certain sarcomas. It is expressed at low levels in normal tissues, except for moderate expression in the testis, ovaries, placenta, adrenal glands, and endometrium [10]. PRAME encodes a membrane-bound protein that elicits an autologous cytotoxic T-cell-mediated immune response. It has also been implicated in immune evasion mechanisms, for example, by interfering with retinoic acid (RA) signaling, a pathway essential for cellular differentiation and apoptosis. Studies suggest that PRAME overexpression inhibits RA-mediated differentiation, growth arrest, and apoptosis by disrupting RA receptor signaling, thereby contributing to tumorigenesis [12]. PRAME overexpression is observed in 88% of primary melanoma tissues and 95% of metastatic tissues [10], and its marked overexpression in melanoma tumor samples has led to its recognition as an immunotherapy target for melanoma treatment [13]. Beyond its therapeutic implications, PRAME has also attracted considerable attention in the diagnostic setting, where its expression profile may provide greater accuracy than conventional markers.

Recent studies report higher sensitivity and specificity compared with traditional markers; however, there is no consensus regarding the optimal cut-off value, with thresholds of 50% or 75% positivity both being proposed [14,15]. Zboras et al. emphasized its diagnostic utility in thin melanomas when lower thresholds are applied [16], and Salih et al. highlighted its role in lentigo maligna margin assessment through a PRAME–Melan-A double-labelling approach [17]. In addition, Cassalia et al. comprehensively reviewed the diagnostic, prognostic, and therapeutic implications of PRAME, underscoring the importance of threshold optimization and inter-laboratory standardization [18]. Given these varying proposals regarding optimal thresholds, a direct comparison of the 50% and 75% cut-offs is warranted to clarify their relative diagnostic performance.

The aim of the present study is to evaluate the diagnostic utility of PRAME immunohistochemistry in distinguishing melanomas from benign melanocytic lesions. We further assess PRAME expression patterns across different melanoma subtypes and compare diagnostic performance at different cut-off thresholds. Our findings may help clarify the role of PRAME in routine diagnostic practice and contribute to establishing standardized interpretation criteria.

## 2. Materials and Methods

### 2.1. Case Selection

A total of 145 melanocytic lesions diagnosed consecutively between 2016 and 2021 were retrospectively retrieved from the archives of our laboratory. The study cohort included 52 melanomas, 27 dysplastic nevi, 23 Spitz nevi, 15 compound nevi, 23 blue nevi, and 5 congenital nevi. Histopathological features were re-evaluated, and representative paraffin-embedded tissue blocks were selected for immunohistochemical analysis.

This study was approved by the local ethics committee (Number: 271, Date: 2 September 2022) and was conducted under the principles of the Helsinki Declaration. Informed consent for participation and publication of data was obtained from all individual participants. Patient anonymity was strictly maintained throughout the study.

### 2.2. Immunohistochemistry

PRAME expression was evaluated in all cases (*n* = 145) using the EP461 rabbit monoclonal antibody (Cell Marque) on formalin-fixed, paraffin-embedded sections processed on an automated stainer (Ventana BenchMark Ultra; Ventana Medical Systems, Tucson, AZ, USA), following the manufacturer’s protocol. Tissue sections were deparaffinized, rehydrated, and subjected to heat-induced antigen retrieval with Cell Conditioning 1 (CC1; high-pH buffer) for 40 min at 98 °C. Endogenous peroxidase activity was blocked with hydrogen peroxide. Detection was performed with the OptiView DAB IHC Detection Kit (Roche Diagnostics, Tucson, AZ, USA), and the primary antibody was applied at a 1:25 dilution. Sebaceous glands served as internal positive controls for PRAME expression.

### 2.3. Evaluation

PRAME expression was independently evaluated by two experienced dermatopathologists who were blinded to the final diagnosis. Inter-rater agreement was quantified using Cohen’s kappa statistic, which demonstrated substantial agreement (κ = 0.81). In cases of scoring discrepancy, the slides were re-examined jointly and consensus was reached through discussion, guided by predefined categorical thresholds. Nuclear staining of melanocytes was considered positive. The proportion of PRAME-positive nuclei was estimated visually (“eyeballing method”), and staining intensity was categorized as: no staining (0), 1–24% (+1), 25–49% (+2), 50–74% (+3), and ≥75% (+4). For comparative analyses between melanoma and nevi, two cut-off thresholds were applied: ≥50% and ≥75%. Statistical analyses, including sensitivity, specificity, and receiver operating characteristic (ROC) curve evaluations, were performed using SPSS version 26.0, with *p*-values < 0.05 considered statistically significant.

## 3. Results

### 3.1. Clinical and Histopathological Results

A total of 145 patients were included in the study, comprising 52.4% males (*n* = 76) and 47.6% females (*n* = 69). The age range of the cohort was 6–93 years, with a mean age of 41.2 years. In terms of anatomical distribution, 56 cases (38.6%) were located on the trunk, 47 (32.4%) on the extremities, 34 (23.4%) on the head and neck, 5 (3.4%) in lymph nodes, and 3 (2.1%) in internal organs. Among primary melanoma cases, the majority were located in the extremities (42.5%, *n* = 17), followed by the head and neck region (32.5%, *n* = 13), and the trunk (25%, *n* = 10). Regarding metastatic melanoma cases, 5 cases (41.7%) were found in lymph nodes, 3 (25%) in internal organs, 2 (16.7%) in the head and neck, 1 (8.3%) in the extremity, and 1 (8.3%) in the trunk (Table 1).

Among the 52 melanoma cases, 12 (23.1%) were metastatic melanoma, 10 (19.2%) were superficially spreading melanoma (SSM), 3 (5,8%) were lentigo maligna melanoma (LMM), 10 (19,2%) were lentigo maligna (LM), 5 (9.6%) were nodular (NM), 5 (9.6%) were acral lentiginous (ALM), and 2 (3.8%) were mucosal melanoma. Typing could not be performed in 5 cases (9.6%).

### 3.2. Immunohistochemical Results

**Figure 1:**
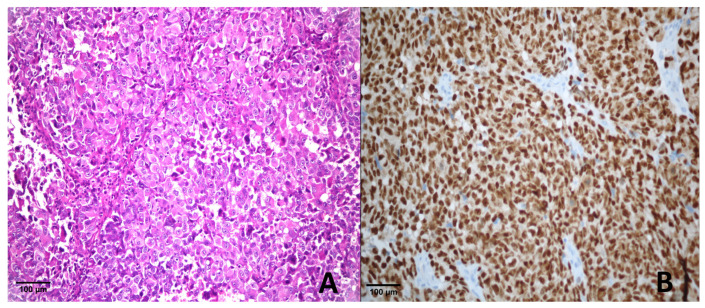
Metastatic melanoma. (**A**) H & E ×400; (**B**) PRAME immunohistochemistry showing strong nuclear positivity (score of +4).

Among the 12 metastatic melanoma cases, 8 exhibited a score of +4 (Figure 1), 2 had a score of +3, and 2 showed a score of +2 for PRAME expression.

**Figure 2:**
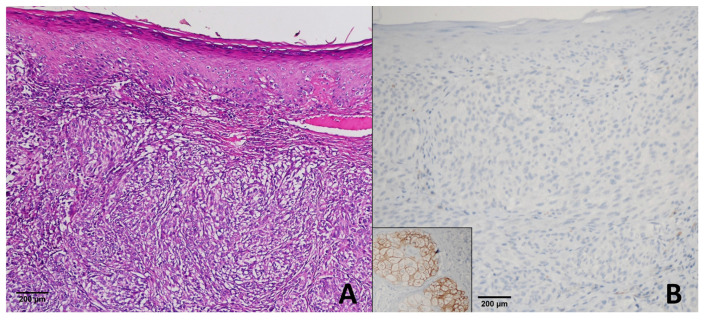
Superficial Spreading Melanoma. (**A**) H & E ×200; (**B**) PRAME immunohistochemistry demonstrating complete absence of nuclear staining in tumor cells (score 0), while sebaceous glands show positive staining and serve as an internal control. This case represents the only PRAME-negative melanoma in our series.

In the 40 primary melanoma cases, 35 demonstrated a score of +4, 3 showed a score of +3, 1 had a score of +2, and 1 was negative (score 0) (Figure 2).

**Figure 3:**
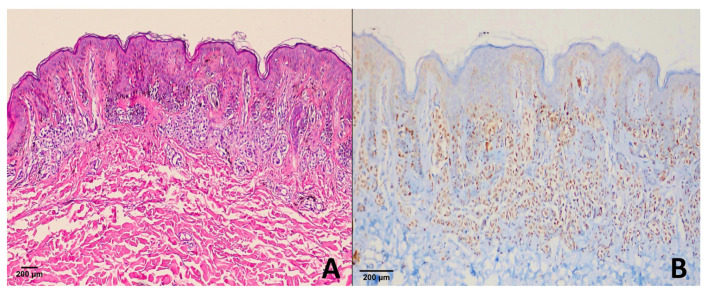
Dysplastic nevus. (**A**) H & E ×100; (**B**) PRAME immunohistochemistry revealing diffuse strong nuclear positivity (score of +4) in melanocytic cells. This represents the exceptional case within our cohort, as most dysplastic nevi were PRAME-negative or only weakly positive.

In the dysplastic nevus group, 1 case showed a score of +4 (Figure 3), 2 cases had a score of +1, and 24 cases were negative (score 0) for PRAME expression.

**Figure 4:**
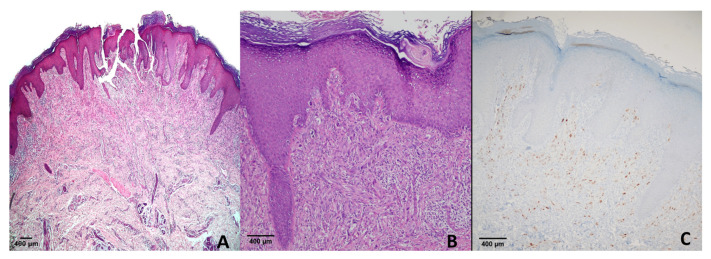
Spitz nevus. (**A**) H & E ×40; (**B**) H & E ×100; (**C**) PRAME immunohistochemistry showing moderate nuclear positivity (score of +3). This case represents one of two atypical Spitz nevi with PRAME expression in our cohort, whereas all conventional Spitz nevi were negative.

All cases of congenital nevi (*n* = 5) and blue nevi (*n* = 23) were negative (score 0). Among 15 compound nevi, 1 case exhibited a score of +1, while the remaining 14 were negative. In 11 Spitz nevus cases, 1 showed a score of +1, and 10 were negative. For atypical Spitz nevi, 2 cases demonstrated a score of +3 (Figure 4), and 10 cases were negative (score 0) (Table 2).

The melanoma case with absent PRAME expression exhibited diffuse S-100 and Melan-A expression, with HMB-45 expression present in both superficial and deep components. Ki-67 labeling index was also elevated. In contrast, the dysplastic nevi showing PRAME 4+ expression (*n* = 1) preserved p16 expression, demonstrated a low Ki-67 index (<5%), and exhibited the typical immunoprofile of benign melanocytic lesions (S-100, Melan-A positive).

#### 3.2.1. Comparison of PRAME Cut-Off Groups in Melanoma and Benign Lesions

A significant difference in PRAME expression was observed between the melanoma group and benign melanocytic lesions. For distinguishing malignant from benign cases, the PRAME ≥ 75% cut-off demonstrated a sensitivity of 82.7% (95% Confidence Interval (CI): 70.3–90.6), a specificity of 98.9% (95% CI: 94.2–99.8), a positive predictive value (PPV) of 97.7%, and a negative predictive value (NPV) of 91.1%. In contrast, the PRAME ≥ 50% cut-off exhibited a sensitivity of 92.3% (95% CI: 81.8–97.0), a specificity of 96.8% (95% CI: 90.9–98.9), a PPV of 94.1%, and an NPV of 95.7% (Table 3).

**Figure 5:**
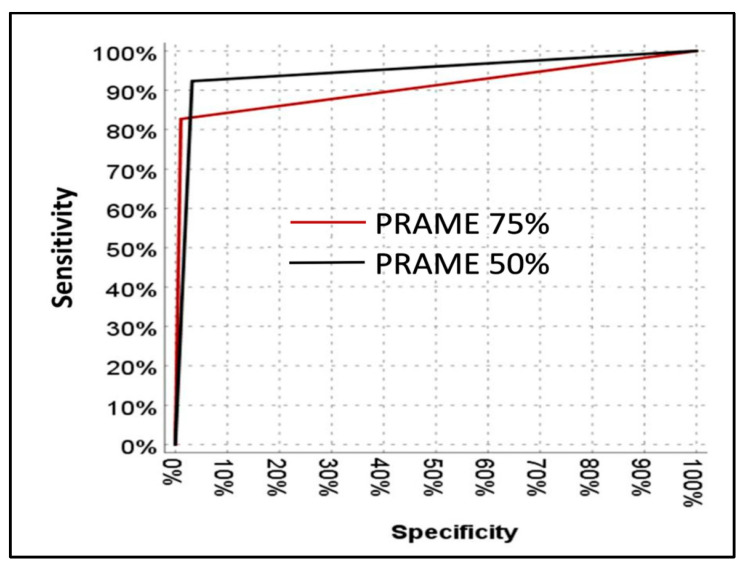
ROC curve showing the diagnostic performance of PRAME expression in distinguishing melanoma from benign melanocytic lesions.

ROC analysis demonstrated high diagnostic accuracy for PRAME at both thresholds, with an AUC of 0.908 (95% CI: 0.845–0.971) for the 75% cut-off and 0.945 (95% CI: 0.898–0.982) for the 50% cut-off (Figure 5). ROC analysis showed that PRAME at both thresholds provided high diagnostic accuracy. The AUC for the 50% cut-off was significantly higher than that for the 75% cut-off (*p* = 0.02).

In our cohort, ROC curve analysis suggested an optimal cut-off of approximately 30%, which is lower than the commonly applied 50% and 75% thresholds. In our cohort, ROC curve analysis suggested an optimal cut-off of approximately 30%, which is lower than the commonly applied 50% and 75% thresholds. This observation is consistent with the findings of Enevoldsen et al., who also identified a 30% digital quantification cut-off to optimize sensitivity and specificity [19]. Nevertheless, because the majority of prior studies have adopted the 50% and 75% thresholds, we focused our analyses on these two cut-offs to facilitate comparability and clinical relevance.

#### 3.2.2. PRAME Immunoreactivity in Melanoma Subtypes

In melanoma cases, PRAME expression of ≥75% was observed in 8 of 12 metastatic melanomas (66.7%), 7 of 10 SSM (70%), 4 of 5 NM (80%), 9 of 10 LM (90%), and in all cases of ALM, LMM and mucosal melanomas (100%). Similarly, PRAME expression of ≥50% melanomas (80%), 9 of 10 LM (90%), and in all cases of NM, ALM, LMM and mucosal melanomas (100%) (Table 4).

#### 3.2.3. Sensitivity and Specificity of PRAME in Melanoma vs. Benign Melanocytic Lesions

In differentiating melanoma from Spitz nevi, PRAME ≥ 75% showed a sensitivity of 82.7% and a specificity of 100%, whereas the ≥50% cut-off increased sensitivity to 92.3% but decreased specificity to 91.3%. For dysplastic nevi, the ≥75% cut-off resulted in a sensitivity of 82.7% and specificity of 96.9%, while the ≥50% threshold improved sensitivity to 92.3%, maintaining specificity at 96.9%. In distinguishing melanoma from compound nevi, PRAME ≥ 75% provided a sensitivity of 82.7% and specificity of 100%, and the ≥50% cut-off increased sensitivity to 92.3% without altering specificity. For blue nevi, both PRAME ≥ 75% and ≥50% cut-offs exhibited high diagnostic accuracy, with sensitivities of 82.7% and 92.3%, respectively, and specificities of 100% in both.

## 4. Discussion

Our study aimed to evaluate the diagnostic utility of PRAME expression in differentiating melanoma from various melanocytic lesions, including dysplastic nevi, congenital nevi, blue nevi, and Spitz nevi. Previous research has underscored the potential of PRAME as a reliable immunohistochemical marker in this context.

### 4.1. Dysplastic Nevi

Lezcano et al. demonstrated that benign nevi generally lack PRAME expression, supporting its role in distinguishing melanomas from benign melanocytic lesions. In their study of 60 dysplastic nevi, no cases showed a PRAME expression of +4; instead, a score of +1 was found in 10 cases and a score of +2 in one case [20]. Similarly, Raghavan et al. reported that none of the 12 dysplastic nevi exhibited a score of +4, with only one case showing 5–10% expression in lesion melanocytes [21]. Chen et al. further confirmed these findings by reporting no +4 PRAME expression in dysplastic nevi, while noting 40% expression in two cases and 5% expression in another two cases [22]. In addition, Gassenmeier et al. observed a score of +4 in one of 35 dysplastic nevi with severe cytological atypia [23], and Cazzato et al. detected diffuse positivity in 4 out of 52 such cases [24]. Our findings align with these previous studies, as we identified a score of +4 in one of 27 dysplastic nevi and a score of +1 in two cases.

In our study, using a 75% threshold to differentiate melanoma from dysplastic nevi resulted in a sensitivity of 82.7% and specificity of 96.9%. Lowering the threshold to 50% increased sensitivity to 92.3% without affecting specificity. These results align with previous reports, where no significant difference in specificity was observed between the two thresholds [23,24].

### 4.2. Congenital, Blue and Benign Nevi

Lezcano et al. included two congenital nevi in their study and reported no staining in these cases [20]. Similarly, in our study, no staining was detected in five congenital nevi. For blue nevi, Lezcano et al. reported no expression in 10 cases [20], while Kline et al. noted a score of +1 in 16 out of 20 cellular blue nevi [25]. Consistent with these findings, our study demonstrated no expression in 23 blue nevus cases. In differentiating melanoma from blue nevi, both 75% and 50% PRAME thresholds yielded a specificity of 100%, though sensitivity increased at the 50% threshold. This supports prior observations that blue nevi rarely show PRAME scores of +3 or +4 [20,25].

Rasic et al. observed no staining in 79.4% of 145 benign nevi, with a score of +1 in 14.9%, a score of +2 in 2.8%, a +3 score in 2.1%, and a +4 score in one case (0.7%) [26]. Lezcano et al. similarly detected low-level expression (+1/+2) in a few benign nevi [20]. In our series, PRAME expression was observed in only one of 15 compound nevi (score +1), all others were negative.

### 4.3. Spitz Nevus

Koh et al. included 10 Spitz nevi and 14 atypical Spitz nevi in their study, reporting a score of +3 in 2 Spitz nevus cases; however, none of the atypical Spitz nevi exhibited a score of +3 or higher [27]. Googe et al. observed no staining in 6 cases of atypical Spitz nevi [28]. In the study by Raghavan et al., staining at or above 60% was detected in 1 of 20 Spitz nevi cases (4%) and in 1 out of 13 atypical Spitz nevi cases (7.7%) [21]. Lezcano et al. reported a score of +4 in 1 of 10 Spitz nevi and a score of +2 or +3 in another case [20]. In our study, 11 Spitz nevi and 12 atypical Spitz nevi were evaluated. Among the Spitz nevi, one case showed a score of +1, whereas in the atypical Spitz nevi, 2 cases exhibited a score of +2, with no staining observed in the remaining cases. Chen et al. noted that none of the Spitz cases showed staining above a 75% threshold, while 89.9% of melanomas were positive [22].

Our data are consistent with those reported by Koh et al. [27], as staining at or above 50% was observed in 2 Spitz nevi, whereas staining at or above 75% was not detected. In differentiating malignant melanoma from Spitz nevi, our study found that using a PRAME threshold of 75% resulted in a sensitivity of 82.7% and a specificity of 100%, while a 50% threshold produced a sensitivity of 92.3% and a specificity of 91.3%. Notably, unlike the other melanocytic lesions evaluated in our study, the use of the 75% threshold for differentiating malignant melanoma from Spitz nevi enhanced specificity.

### 4.4. Melanoma

#### 4.4.1. Metastatic Melanoma

Several studies have demonstrated high PRAME expression in metastatic melanoma. Gradecki et al. reported PRAME expression in 97.4% of cases (151/155), with 41.3% showing a score of +4, 29.7% a score of +3, 11.6% a score of +2, and 14.8% a score of +1 [29]. Notably, when applying a 50% cut-off, the rate of strong positivity increased to 81%. Similarly, Lezcano et al. observed a score of +4 in 92% of 100 metastatic melanoma cases [20], while Kaczorowski et al. reported PRAME expression in 75% of 248 cases using an 80% threshold [30].

In our cohort, PRAME expression was detected in all metastatic melanoma cases. Among 12 cases, 66.7% exhibited a score of +4, 16.7% a score of +3, and 16.7% a score of +2. The sensitivity was 66.7% at the +4 threshold, increasing to 83.3% at the +3 threshold. Variability in reported positivity rates across studies may be attributed to differences in immunohistochemical protocols, scoring criteria, and antibody clones used. While most prior studies utilized the EPR20330 clone, our study employed the EP461 clone. Interestingly, Kaczorowski et al., who also used the EP461 clone, reported a score of +4 in 75% of metastatic melanoma [30], which aligns more closely with our findings.

#### 4.4.2. Primary Melanoma

##### Mucosal Melanoma

Scheurleer et al. investigated PRAME expression in sinonasal mucosal melanomas and reported positivity in all cases [31]. Similarly, another study comprising 24 cases found that 4 cases exhibited staining of 30% or lower, while the remaining 20 cases showed staining of 55% or higher [32]. In our study, two mucosal melanoma cases were included, both of which demonstrated a score of +4, aligning with findings from the literature. Given the very limited number of cases, however, these observations should be regarded as exploratory and interpreted with caution.

##### Cutaneous Melanoma Subtypes

Chen et al. investigated PRAME expression in 178 primary melanomas, reporting positivity in 160 cases (89.9%). The distribution of PRAME expression among melanoma subtypes was: 100% in SSM, 80% in LMM, 91.4% in NM, and 87.8% in ALM [22]. Similarly, Cazzato et al. detected PRAME expression in 104 out of 127 cases (81.8%), with high expression rates across subtypes [24]. Lezcano et al. reported a score of +4 in 92.5% of SSM, 88.6% of LMM, 94.4% of ALM, and 90% of NM cases [20].

In our cohort, PRAME expression was observed in 39 out of 40 primary melanoma cases (97.5%), with one SSM case showing no expression. Specifically, 7 out of 10 SSM cases (70%) had a score of +4, and 1 case (10%) had a score of +3. Four out of 5 NM cases (80%) showed a score of +4, and 1 (20%) had a score of +3. All ALM, LMM, mucosal, and in situ melanoma cases (100%) demonstrated a score of +4. Compared to prior reports, PRAME expression rates in our SSM and NM subtypes were slightly lower, while ALM cases aligned with Lezcano et al. [20], and LMM cases showed higher positivity. However, the limited number of cases may affect comparability, highlighting the need for larger series.

### 4.5. Threshold Selection

Many studies in the literature have used a 75% threshold to define PRAME expression [23,28,33,34]. However, several authors have proposed lower thresholds to improve sensitivity. For instance, Rawson et al. reported that applying a 50% cut-off increased the overall positivity rate to 70% when combining scores of +3 and +4, compared to a 38% positivity rate at the +4 level alone [35]. Similarly, Raghavan et al. utilized a 60% threshold, achieving higher sensitivity [21] (Table 5).

In our study, we compared the diagnostic performance of both the 50% and 75% thresholds. Lowering the cut-off to 50% improved sensitivity from 82.7% to 92.3%, with only a slight decrease in specificity (from 98.9% to 96.8%). These results suggest that a 50% threshold provides a more practical balance between sensitivity and specificity for differentiating melanoma from benign melanocytic lesions. Nevertheless, when evaluating Spitz nevi specifically, the 75% threshold yielded higher specificity, as two cases exhibited a score of +3.

Another factor contributing to variability across studies is the choice of PRAME antibody clone. Most previous research has employed the EPR20330 clone, whereas our study used the EP461 clone, for which available data remain limited. Kaczorowski et al., who also used the EP461 clone, defined ≤5% staining as negative and ≥80% as diffuse positivity. They reported PRAME expression in 99% of mucosal melanomas and 87% of metastatic melanomas, with diffuse positivity in 85% and 75% of cases, respectively. However, detailed data regarding expression percentages above the 50% threshold were not provided in their study [30].

Overall, the literature shows considerable variability in PRAME sensitivity and specificity. For example, Rasic et al. reported sensitivity of 73.6% and specificity of 96.5% using a 75% cut-off [26]. O’Connor et al. found that reducing the threshold from 75% to 50% increased sensitivity from 63% to 74%, though specificity decreased from 97% to 91%. Based on their findings, they suggested categorizing PRAME expression into diagnostic ranges: ≤25% favoring benign nevi, 26–75% as indeterminate, and >75% suggestive of melanoma [15]. Similarly, Lohman et al. observed improved sensitivity at a ≥50% threshold [14]. Although a universal consensus regarding the optimal cut-off is lacking, many subsequent studies have adopted the 75% threshold based on the widely cited work of Lezcano et al. [20].

While lowering the cut-off improved sensitivity, it is important to acknowledge that this approach could potentially increase the risk of overdiagnosing melanoma, particularly in lesions with borderline histopathologic features, such as dysplastic nevi or certain Spitz nevi. In our cohort, one SSM was entirely PRAME-negative, whereas a subset of dysplastic nevi demonstrated diffuse positivity (+4). These paradoxical findings, also reported in previous studies [20,24], may be explained by factors such as tumor heterogeneity, clonal variation, or epigenetic alterations. Such exceptions highlight that PRAME immunostaining should not be interpreted in isolation, but rather integrated with the overall histopathologic and clinical context. Moreover, the clinical benefit of a higher sensitivity cut-off would ideally be validated with long-term follow-up studies to ensure that increased detection translates into improved patient outcomes. Future multicenter studies with larger sample sizes and outcome data are warranted to refine threshold recommendations.

### 4.6. Limitations

Our study has several limitations. First, the relatively small sample size may affect the broader applicability of the findings. In particular, analyses of certain melanoma subtypes (e.g., mucosal melanoma, and lentigo maligna) and Spitz nevus are based on very few cases and should therefore be considered exploratory. Second, the proportion of PRAME expression was estimated visually (“eyeballing method”), which may introduce observer variability. Although this approach is widely used in routine practice, digital image analysis has recently been proposed as a more objective and reproducible alternative [19], and should be considered in future studies to improve consistency and standardization. Third, this was a retrospective single-center study, which may limit generalizability. Fourth, only one antibody clone (EP461) was used, whereas most other studies employed the EPR20330 clone, potentially affecting inter-study comparability. Finally, outcome data and clinical follow-up were not available, precluding prospective validation of the diagnostic cut-offs.

## 5. Conclusions

Our study supports PRAME as a useful marker in distinguishing melanomas from benign melanocytic lesions. Applying a 50% threshold for PRAME expression improves sensitivity while maintaining high specificity. However, PRAME immunoreactivity should be interpreted with caution, as certain exceptions may occur. Notably, we observed a score of +4 in one dysplastic nevus case, absence of staining in one SSM case, and variable expression in Spitz nevi, underscoring that PRAME alone is not entirely definitive. Therefore, histomorphological evaluation remains the cornerstone in the diagnosis of melanocytic lesions. Taken together, our findings underscore that PRAME should be regarded as an adjunctive tool rather than a stand-alone diagnostic marker, and its interpretation must always be contextualized with morphological and clinical features.

Future multicenter studies with larger sample sizes, standardized protocols, integration of digital quantification tools, and correlation with long-term patient outcomes are warranted to refine PRAME interpretation and to establish more robust and reproducible diagnostic thresholds.

## Figures and Tables

**Table 1:** Clinical and histopathological features of the cases included in our study.

Age Range		6.0–93.0
Average Age		41.2
**Gender**	Female	69/145
	Male	76/145
**Diagnosis**	Primary malignant melanoma	40/145
	Metastatic Melanoma	12/145
	Dysplastic Nevus	27/145
	Blue Nevus	23/145
	Compound Nevus	15/145
	Spitz Nevus	23/145
	Congenital Nevus	5/145
**Location**	Trunk	56/145
	Extremity	47/145
	Head and Neck	34/145
	Lymph node	5/145
	Internal organs	3/145

**Table 2:** PRAME Score Distribution.

Score	Metastatic Melanoma	Melanoma (Primary)	Dysplastic Nevus	Congenital Nevus	Compound Nevus	Blue Nevus	Spitz Nevus	Atypical Spitz Nevus
+4	8/12	35/40	1/27	0/5	0/15	0/23	0/11	0/12
+3	2/12	3/40	0/27	0/5	0/15	0/23	0/11	2/12
+2	2/12	1/40	0/27	0/5	0/15	0/23	0/11	0/12
+1	0/12	0/40	2/27	0/5	1/15	0/23	1/11	0/12
0	0/12	1/40	24/27	5/5	14/15	23/23	10/11	10/12

**Table 3:** Diagnostic performance of PRAME at different cut-off thresholds (50% vs. 75%), including sensitivity, specificity, PPV, NPV, and *p*-values.

		Benign	Malignant	Sensitivity	PPV	Specificity	NPV	*p*
PRAME	<75%	92	9	82.7%	97.7%	98.9%	91.1%	<0.001
	≥75%	1	43					
PRAME	<50%	90	4	92.3%	94.1%	96.8%	95.7%	<0.001
	≥50%	3	48					

**Table 4:** Comparison of PRAME Expression Rates at 75% and 50% Cut-offs.

	PRAME ≥ 75%	PRAME ≥ 50%
Metastatic melanoma	8/12 (66.7%)	10/12 (83.3%)
Superficial spreading melanoma	7/10 (70%)	8/10 (80%)
Nodular melanoma	4/5 (80%)	5/5 (100%)
Acral lentiginous melanoma	5/5 (100%)	5/5 (100%)
Lentigo maligna melanoma	3/3 (100%)	3/3 (100%)
Lentigo maligna (in situ)	9/10 (90%)	9/10 (90%)
Mucosal melanoma	2/2 (100%)	2/2 (100%)

**Table 5:** Reported sensitivity and specificity of PRAME immunohistochemistry for distinguishing melanoma from benign melanocytic lesions in previous studies.

Study	Threshold (%)	Sensitivity (%)	Specificity (%)	Clone
Lezcano et al. [20]	75	83	100	EPR20330
Raghavan et al. [21]	60	92	98	EPR20330
Alomari et al. [34]	75	53	86	EPR20330
Googe et al. [28]	75	79	99	EPR20330
Gassenmaeier et al. [23]	75	59	98	QR005
Lohman et al. [14]	75	67	100	EPR20330
Lohman et al. [14]	50	75	97	EPR20330
Gradecki et al. [29]	75	41.3	-	EPR20330
Gradecki et al. [29]	50	81	-	EPR20330
See et al. [36]	50	94	100	EPR20330
Kaczorowski et al. [30]	80	75	-	EP461
Our study	75	82.7	98.9	EP461
Our study	50	92.3	96.8	EP461

## Data Availability

All data analyzed in the present study are included in this article.

## Abbreviations

The following abbreviations are used in this manuscript:

ALM	Acral Lentiginous Melanoma
CI	Confidence Interval
H & E	Hematoxylin and Eosin
LM	Lentigo Maligna
LMM	Lentigo Maligna Melanoma
NM	Nodular Melanoma
NPV	Negative Predictive Value
PRAME	Preferentially Expressed Antigen in Melanoma
PV	Positive Predictive Value
RA	Retinoic Acid
SSM	Superficial Spreading Melanoma
